# Optical control of GPR40 signalling in pancreatic β-cells†
†Electronic supplementary information (ESI) available. See DOI: 10.1039/c7sc01475a


**DOI:** 10.1039/c7sc01475a

**Published:** 2017-08-30

**Authors:** James Allen Frank, Dmytro A. Yushchenko, Nicholas H. F. Fine, Margherita Duca, Mevlut Citir, Johannes Broichhagen, David J. Hodson, Carsten Schultz, Dirk Trauner

**Affiliations:** a Department of Chemistry , Center for Integrated Protein Science , Ludwig Maximilians University Munich , Butenandtstraße 5-13 , 81377 Munich , Germany; b European Molecular Biology Laboratory (EMBL) , Cell Biology & Biophysics Unit , Meyerhofstraße 1 , 69117 Heidelberg , Germany . Email: schultz@embl.de; c Institute of Organic Chemistry and Biochemistry , Academy of Sciences of the Czech Republic , Flemingovo namesti 2 , 16610 Prague 6 , Czech Republic; d Institute of Metabolism and Systems Research (IMSR) , University of Birmingham , Birmingham , B15 2TT , UK . Email: d.hodson@bham.ac.uk; e Centre for Endocrinology, Diabetes and Metabolism , Birmingham Health Partners , Birmingham , B15 2TH , UK; f COMPARE University of Birmingham and University of Nottingham Midlands , UK; g Department of Chemistry , University of Milan , Via Golgi 19 , 20133 , Milan , Italy; h Max-Planck Institute of Medical Research , Jahnstr. 29 , 69120 Heidelberg , Germany; i Dept. of Physiology and Pharmacology , Oregon Health and Science University , Portland , OR 97237 , USA; j Department of Chemistry , New York University , 100 Washington Square East , New York , NY 10003-6699 , USA . Email: dirktrauner@nyu.edu

## Abstract

Fatty acids activate GPR40 and K^+^ channels to modulate β-cell function. Herein, we describe the design and synthesis of **FAAzo-10**, a light-controllable GPR40 agonist based on Gw-9508. **FAAzo-10** is a potent GPR40 agonist in the *trans*-configuration and can be inactivated on isomerization to *cis* with UV-A light. Irradiation with blue light reverses this effect, allowing **FAAzo-10** activity to be cycled ON and OFF with a high degree of spatiotemporal precision. In dissociated primary mouse β-cells, **FAAzo-10** also inactivates voltage-activated and ATP-sensitive K^+^ channels, and allows us to control glucose-stimulated Ca^2+^ oscillations in whole islets with light. As such, **FAAzo-10** is a useful tool to study the complex effects, with high specificity, which FA-derivatives such as Gw-9508 exert at multiple targets in mouse β-cells.

## Abstract 2

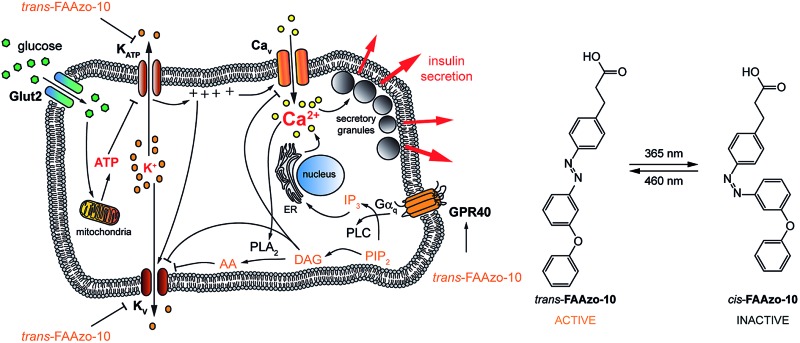
Fatty acids activate GPR40 and K^+^ channels to modulate β-cell function.

## Introduction

Although minimalistic in structure and often viewed as subunits of more complex lipids or simply an energy source, fatty acids can have profound effects on cell signalling.^1^–^4^ Free fatty acids most often consist of a long, unbranched carbon chain attached to a carboxyl headgroup, which is largely deprotonated and thus negatively charged at physiological pH.^5^ They are amphiphilic molecules with diverse structures that vary in the chain length and the level of unsaturation. A number of transmembrane signalling proteins, including G protein-coupled receptors (GPCRs) such as GPR40,^6^ are stimulated by free fatty acids,^7^ resulting in a rise in the intracellular Ca^2+^ concentration ([Ca^2+^]_i_) in insulin-secreting pancreatic β-cells through activation of phospholipase C.^8^–^10^ Given the role of GPR40 in glucose homeostasis, synthetic agonists for these receptors such as Gw-9508 (ref. 11 and 12) and TAK-875 (ref. 13 and 14) have received significant attention as potential treatments for type 2 diabetes mellitus.^15^,^16^ However, a phase III clinical trial for TAK-875 was recently terminated due to off-target effects and toxicity concerns.^17^,^18^

**Fig. 1:**
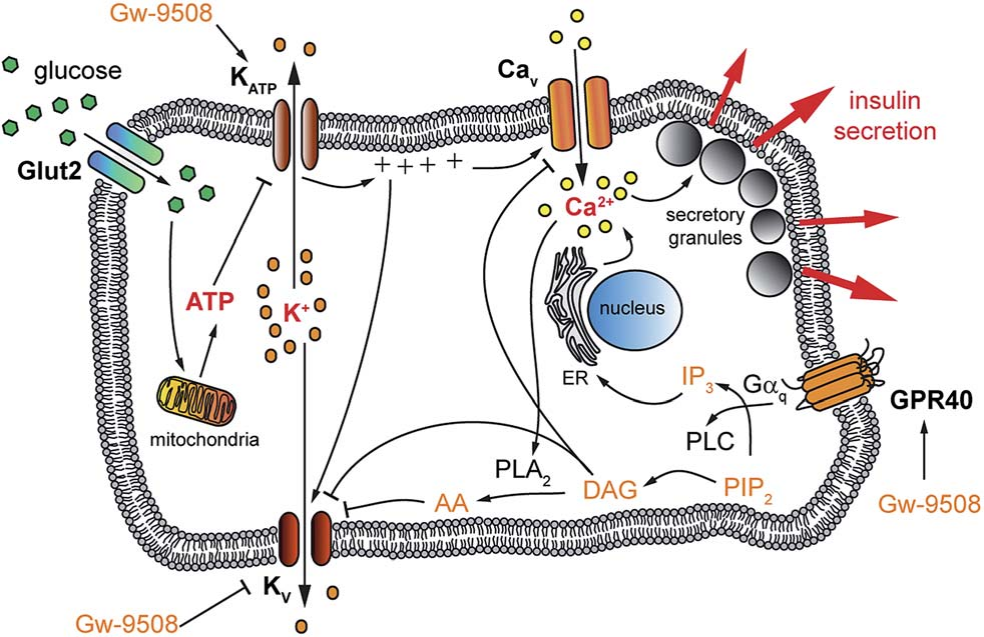
Glucose-stimulated insulin secretion (GSIS) from pancreatic β-cells. Upon uptake into the pancreatic β-cell, glucose is metabolized into ATP. The rising ATP/ADP ratio inhibits K_ATP_ which causes membrane depolarization and the opening of Ca_v_. The resulting increased [Ca^2+^]_i_ triggers the fusion of secretory granules and the release of insulin. K_v_ channels work to repolarize the cell, generating oscillations in [Ca^2+^]_i_. GPR40 stimulation also leads to increased [Ca^2+^]_i_, further potentiating GSIS.

Glucose-stimulated insulin secretion (GSIS) relies on transport of glucose into the β-cell, followed by its metabolism to ATP. The resulting increase in the ATP/ADP ratio leads to closure of ATP-sensitive K^+^ channels (K_ATP_) and subsequent membrane depolarization. This causes the opening of voltage activated L-type Ca^2+^ channels (Ca_v_) and an increase in [Ca^2+^]_i_, driving exocytosis of insulin secretory granules.^19^ Subsequent activation of delayed rectifier voltage-activated K^+^ (K_v_) channels leads to repolarization of the membrane, reduced Ca^2+^ entry through Ca_v_ channels and termination of insulin secretion (Fig. 1).^20^ This is complemented by the action of other messengers, including those stemming from GPCRs (so-called “amplifying” signals). Notably, the amplifying effects of GPR40 activation on insulin secretion remain elusive due to conflicting results in different experimental conditions,^12^,^21^ which could be attributed to effects of FAs at different targets. For example, fatty acids are known to directly affect various K^+^ channels that are involved in modulation of the [Ca^2+^]_i_ oscillation frequency,^1^,^22^,^23^ demonstrating their complex pharmacology and vital role in β-cell signalling. Therefore, a tool that could enable precise control over GPR40 signalling may be useful to better understand the effects of fatty acids, as well as specific agonists, on β- and other cell functions. This could lead to the development of novel therapeutics by delineating the receptor conformations required for biased signalling.^18^,^24^

Previous studies in our laboratories have focused on the development of photoswitchable sulfonylureas and incretins, with which we could place pancreatic β-cell function under the precise spatiotemporal control of light.^25^–^29^ We also showed that photoswitchable diacylglycerols^30^–^32^ affect β-cell [Ca^2+^]_i_ and insulin secretion. These diacylglycerols were constructed from a photoswitchable fatty acid (FAAzo) chain, however the pharmacology of these FAAzos alone remains largely unexplored. Given the sensitivity of GPR40 to unsaturated, and sometimes aryl-containing free fatty acid-like molecules, we hypothesized that the FAAzos themselves could enable optical control of this GPCR. Herein, we describe a novel approach towards the optical control of fatty acid/GPR40 signalling in β-cells.

## Results and discussion

**Fig. 2:**
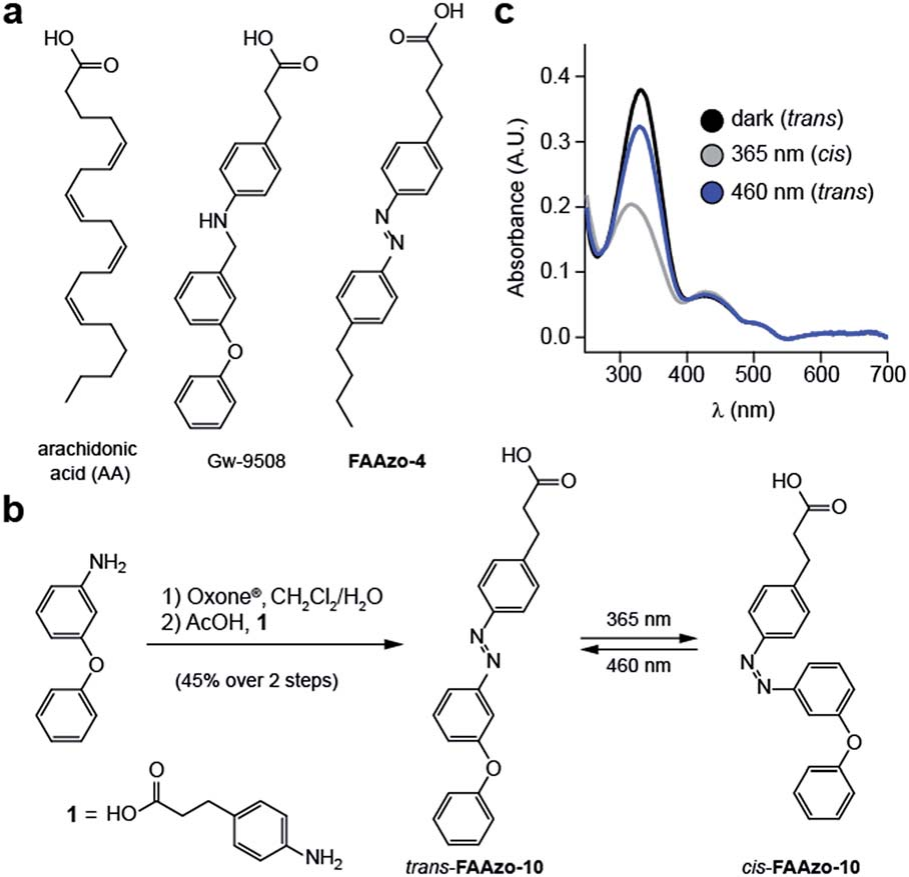
Design and synthesis of photoswitchable GPR40 agonists. (a) The chemical structures of Gw-9508, AA and **FAAzo-4**. (b) Chemical synthesis of **FAAzo-10**, a photoswitchable derivative of Gw-9508. (c) The UV-Vis spectra of **FAAzo-10** in its dark-adapted (black), UV-adapted (gray) and blue-adapted (blue) states (20 μM in PBS).

Although GPR40 is activated by long-chain fatty acids such as arachidonic or linoleic acid,^10^ various aryl-containing carboxylic acids such as Gw-9508 are known to produce a similar effect (Fig. 2a).^3^ We recognized that the benzyl-aniline moiety of Gw-9508 could be easily substituted by a phenyl diazene, and would afford a photoswitchable ligand with little disturbance to the overall size and structure of the drug. Therefore, we synthesized the azologue^33^ of Gw-9508, **FAAzo-10**, using the Mills reaction after nitroso formation in two steps and 45% overall yield (Fig. 2b). Similar to the other members of the FAAzo family,^30^**FAAzo-10** behaved as a regular azobenzene and could be isomerized between its thermally stable *trans*-form to the *cis*-form with UV-A light (Fig. 2c). The process could be reversed by irradiation with blue light, and photoswitching could be repeated over many cycles.

**Fig. 3:**
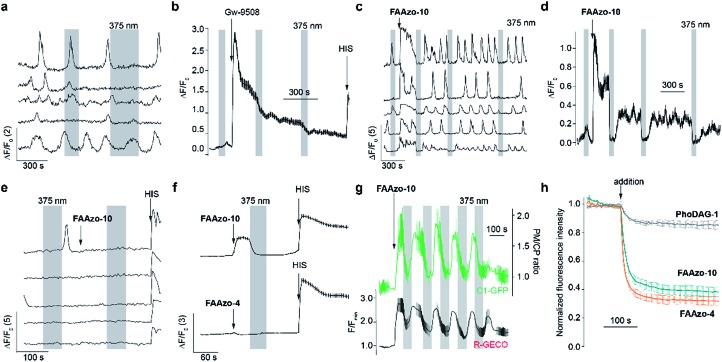
FAAzos enable optical control of GPR40 in HeLa cells expressing GPR40, the diacylglycerol sensor C1-GFP and the genetically encoded [Ca^2+^]_i_ sensor R-GECO. (a) Spontaneous oscillations of [Ca^2+^]_i_ were observed before addition of any compound. (b) Gw-9508 (200 nM) caused an increase in [Ca^2+^]_i_ that was not affected by 375 nm irradiation. HIS (10 nM) application caused an increase in [Ca^2+^]_i_ (*n* = 179 cells from two experiments). (c, d) *trans*-**FAAzo-10** (200 nM) increased [Ca^2+^]_i_, and isomerization to *cis*-**FAAzo-10** with 375 nm light reversed this effect. Displayed as (c) individual [Ca^2+^]_i_ traces from representative cells and (d) the average [Ca^2+^]_i_ for many cells (*n* = 157 cells from two experiments). (e) In cells not expressing GPR40, **FAAzo-10** (200 nM) did not affect [Ca^2+^]_i_. (f) At 200 nM, **FAAzo-4** (*n* = 211 cells from two experiments) did not affect [Ca^2+^]_i_ when compared to **FAAzo-10** (*n* = 153 cells from two experiments). (g) C1-GFP translocated to the plasma membrane alongside an increase in [Ca^2+^]_i_ when stimulated by *trans*-**FAAzo-10** (20 μM, *n* = 10 cells from one representative experiment). Translocation (green) is displayed as the plasma membrane to cytoplasm (PM/CP) C1-GFP fluorescence intensity ratio. (h) Quantification of cell entry using fluorescence quenching of coumaryl-AA-loaded (100 nM) HeLa cells after application (100 nM, 2 experiments each) of **FAAzo-4** (*n* = 29 cells, orange), **FAAzo-10** (*n* = 23 cells, green) and **PhoDAG-1** (*n* = 39 cells, grey), respectively. Error bars were calculated as ±s.e.m.

We then characterized the effects of **FAAzo-10** on GPR40 in HeLa cells using confocal fluorescence microscopy and the genetically encoded fluorescent [Ca^2+^]_i_ reporter R-GECO.^34^ When transiently transfected with GPR40, a portion of cells displayed spontaneous [Ca^2+^]_i_ oscillations without the addition of any external stimuli (Fig. 3a and S1a†). Gw-9508 induced a GPR40-dependent increase in the rate and intensity of [Ca^2+^]_i_ oscillations, that was not affected by UV-A-irradiation (Fig. 3b and S1b†). In cells without GPR40, no response was observed (Fig. S1c and d†). Complementary to this result, the application of *trans*-**FAAzo-10** (200 nM) stimulated a significant increase in [Ca^2+^]_i_ in HeLa cells expressing GPR40 (Fig. 3c and d). On isomerization to *cis* with 375 nm irradiation, a sharp decrease in the [Ca^2+^]_i_ was observed. The effect was reversed and [Ca^2+^]_i_ increased on termination of the irradiation. In cells lacking GPR40, **FAAzo-10** did not affect [Ca^2+^]_i_ (Fig. 3e and S1e†). We also evaluated the effect of **FAAzo-4**, which possesses a similar structure to **FAAzo-10**, but was not active at this low concentration (Fig. 3f). Histamine^35^ (HIS, 10 μM) was used as a positive control and triggered a large increase in [Ca^2+^]_i_, independent of GPR40 expression (Fig. 3 and S1†).

To investigate the downstream effects of GPR40 activation, we expressed the fluorescent diacylglycerol reporter C1-GFP, which translocates to the plasma membrane in response to increased diacylglycerol levels following PLC activation.^36^ Gw-9508 (200 nM) triggered C1-GFP translocation towards the plasma membrane, indicating activation of the GPCR (Fig. S1f and g†). On application of *trans*-**FAAzo-10** (20 μM), we observed a similar effect on C1-GFP translocation. This could be reversed following isomerization to *cis*-**FAAzo-10** with 375 nm irradiation, and translocation could be repeated over many cycles (Fig. 3g). These results demonstrate that oscillations in GPR40 activity and its downstream effectors (*i.e.* PLC, [Ca^2+^]_i_ and diacylglycerols) can be modulated with good temporal control.

Surprisingly, the effects induced by the FAAzos in HeLa cells did not diminish over time (Fig. 3), unlike those induced by the photoswitchable diacylglycerol **PhoDAG-1**, which decreased in magnitude over multiple UV-A pulses of the same length.^31^ To control for differences in cell loading, we applied the coumarinyl-ester of AA (cg-AA) to the HeLa cells.^6^ This fluorescent fatty acid-derivative localizes predominantly at the inner cellular membranes.^31^ By monitoring the quenching of coumarin fluorescence by the azobenzene of FAAzos, we demonstrated that this observed variance in activity was not due to variable FAAzo uptake by cells. Application of both FAAzos caused a rapid and large (>60%) decrease in coumarin fluorescence (Fig. 3h), especially when compared to the quenching effect of **PhoDAG-1** (<20%), which is known to remain trapped on the outer plasma membrane.^31^ A cellular lipid analysis by thin layer chromatography (TLC) confirmed only minor FAAzo metabolism in cells incubated with **FAAzo-4** and **FAAzo-10** (100 μM) for up to 1 h (Fig. S2†). Together, these results demonstrate that the FAAzos are quickly taken up into cells, and only minimally metabolized over the timeframe of a typical imaging experiment.

**Fig. 4:**
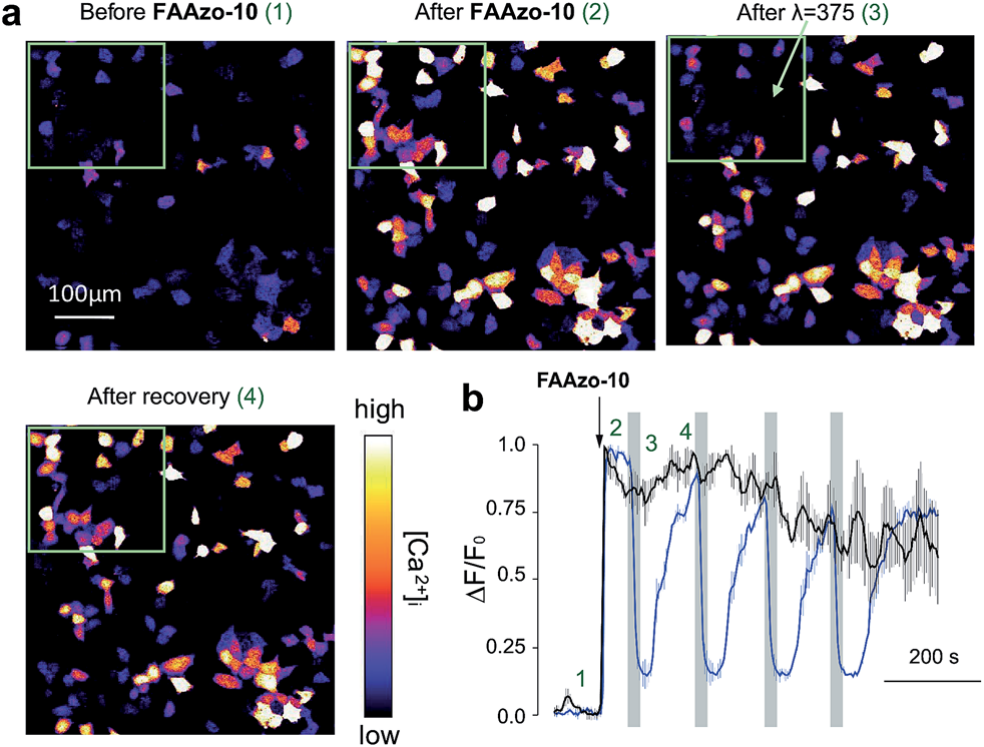
Spatial control of GPR40 signalling with **FAAzo-10**. (a) Confocal images of HeLa cells expressing GPR40 and R-GECO before and after treatment with **FAAzo-10** (200 nM) and illumination with 375 nm light. The green rectangle indicates the area of illumination. After addition of **FAAzo-10**, all transfected cells showed increased [Ca^2+^]_i_. Following illumination, only cells within the green rectangle showed a sharp decrease in [Ca^2+^]_i_ levels, which recovered after termination of illumination. Scale bar = 100 μm. (b) Normalized [Ca^2+^]_i_ in illuminated cells (within the green rectangular in (a)) in blue (*n* = 52) and those in unilluminated cells (outside the green rectangular) in black (*n* = 82). Time points 1–4 correspond to the respective time frames in (a). Error bars were calculated as ±s.e.m.

A major advantage of **FAAzo-10** when compared to conventional agonists is the ability to modulate GPR40 activity with increased spatial precision. By illuminating only cells of interest, we were able to selectively control GPR40 activity without affecting signalling in neighbouring unilluminated cells (Fig. 4). This allows GPR40 activity to be controlled in a spatially defined manner in large patches of cells or complex tissues.

**Fig. 5:**
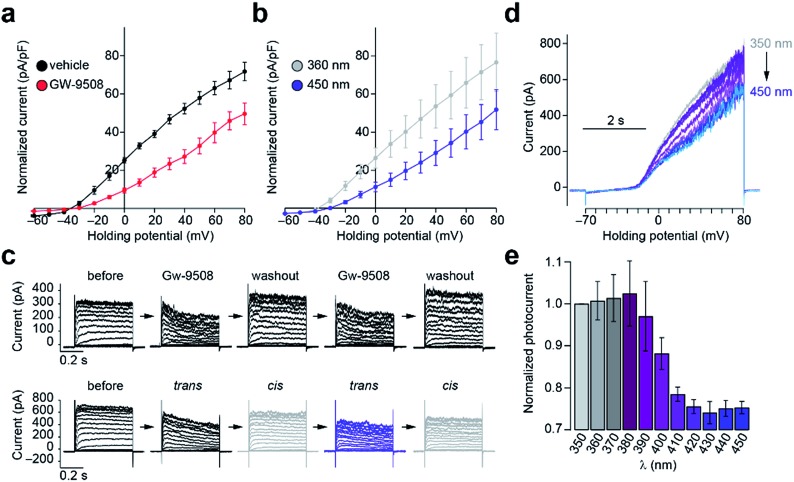
Optical control of β-cell K_v_ channel activity. The whole-cell K_v_ channel current in dissociated wt mouse β-cells was measured using patch clamp electrophysiology. (a) An *IV*-plot showed that Gw-9508 (50 μM) (*n* = 8 cells from 2 animals) reduced the K_v_ conductance when compared to a vehicle control (*n* = 6 cells from 3 animals). (b) Under blue light, *trans*-**FAAzo-10** (20 μM) reduced the whole-cell K_v_ current. Isomerization to *cis*-**FAAzo-10** with UV-A light reversed this effect (*n* = 7 cells from 3 animals). (c) Similar to the wash-in and wash-out of Gw-9508, **FAAzo-10** could be activated and inactivated over several cycles using irradiation. Shown are *IV*-steps from –70 to +80 mV from representative cells. (d, e) An action spectrum between 350–450 nm showed that K_v_ activity could be fine-tuned by changing the irradiation wavelength. Displayed as (d) overlaid sequential voltage ramps (–70 to +80 mV) from a representative cell and (e) the normalized current (to *I*_350 nm_) under each wavelength (*n* = 3 cells from 2 animals). Error bars were calculated as ±s.e.m.

To evaluate the effects of **FAAzo-10** on K^+^ channels, we used whole-cell electrophysiology in dissociated mouse β-cells, which express both K_v_ and K_ATP_ channels.^37^,^38^ K_v_ channel conductance is a major determinant of the [Ca^2+^]_i_ oscillation frequency.^20^ Like AA^31^ and Gw-9508 (Fig. 5a), *trans*-**FAAzo-10** reduced K_v_ channel conductance in the dark or under blue irradiation (Fig. 5b). On isomerization to *cis*-**FAAzo-10**, K_v_ channel activity was restored to a level comparable with the vehicle controls (Fig. 5a). **FAAzo-10** could be switched ON and OFF repeatedly, effectively allowing us to quickly mimic the wash-in and wash-out of Gw-9508 using only a UV-A/blue irradiation (Fig. 5c). Furthermore, we could also fine-tune the effect of **FAAzo-10** with greater precision by scanning through different irradiation wavelengths. The K_v_ conductance could be precisely controlled by gradually increasing the blocking effect of **FAAzo-10** when scanning from UV-A to blue wavelengths. This was demonstrated by applying voltage ramps under 350–450 nm irradiation (Fig. 5d and e).

**Fig. 6:**
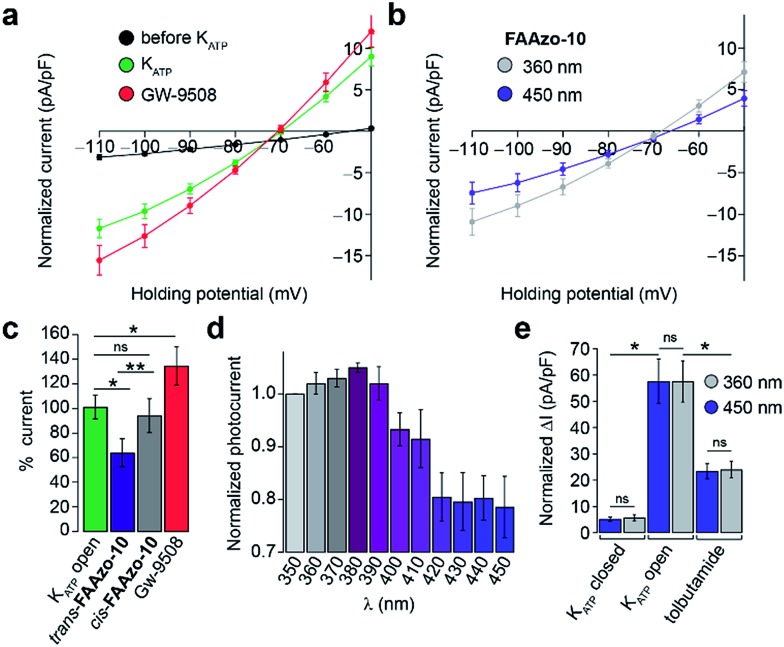
Optical control of β-cell K_ATP_ channels. The whole-cell K_ATP_ current from dissociated mouse β-cells was measured between –110 to –50 mV. (a–c) After dialysis of the cytoplasm with the pipette solution, the K_ATP_ current developed to a steady state (black = before, *n* = 21; green = after, *n* = 20 cells from 2 animals). Application of Gw-9508 (20 μM, red, *n* = 9 cells from 2 animals) increased K_ATP_ conductance. In contrast, the application of *trans*-**FAAzo-10** (20 μM, blue) decreased the K_ATP_ current, while isomerization to *cis*-**FAAzo-10** (gray) reversed this effect (*n* = 7 cells from 2 animals). Data is displayed as (a, b) the full *IV* relationship between –110 to –50 mV and (c) the % K_ATP_ current (at –110 mV) for multiple cells, normalized to the K_ATP_ open (green) state. (d) In the presence of **FAAzo-10**, an action spectrum between 350–450 nm revealed that K_ATP_ was inhibited the most under blue irradiation. Irradiation with UV-A light prevented **FAAzo-10** from blocking the K_ATP_ current. Displayed as the normalized current (to *I*_350 nm_) under each wavelength (*n* = 3 cells from one animal). (e) UV-A or blue irradiation alone did not affect the K_ATP_ current, and tolbutamide (40 μM) significantly reduced the magnitude of the K_ATP_ current (Δ*I* from –110 to –50 mV, *n* = 3 cells from one animal). ns = *P* > 0.05, *P < 0.05, **P < 0.01. Error bars were calculated as ±s.e.m.

Gw-9508 has also been shown to potentiate K_ATP_ channels in mouse β-cells.^11^ We measured the whole-cell K_ATP_ current without extracellular glucose. *IV*-curves were measured between –110 and –50 mV to exclude any effect of the K_v_ channels. After dialysis of the cytoplasm with intracellular buffer to reduce the ATP/ADP ratio, the K_ATP_ current increased to a steady state (Fig. 6a and S3a†). In line with previous reports, Gw-9508 increased the K_ATP_ conductance further (Fig. 6a and c).

Interestingly, *trans*-**FAAzo-10** behaved differently, and reduced the K_ATP_ conductance, while isomerization to *cis*-**FAAzo-10** reversed the effect (Fig. 6b). Similar to the effects observed on K_v_ channels, **FAAzo-10** activity at K_ATP_ could be fine-tuned by altering the irradiation wavelength (Fig. 6d). Under blue irradiation, the K_ATP_ current was reduced, while the blockade was reversed towards UV-A wavelengths. In control experiments, application of the sulfonylurea tolbutamide reduced the K_ATP_ current significantly (Fig. 6e and S3a†), and neither UV-A nor blue irradiation alone affected the K_ATP_ conductance (Fig. 6e and S3†).

**Fig. 7:**
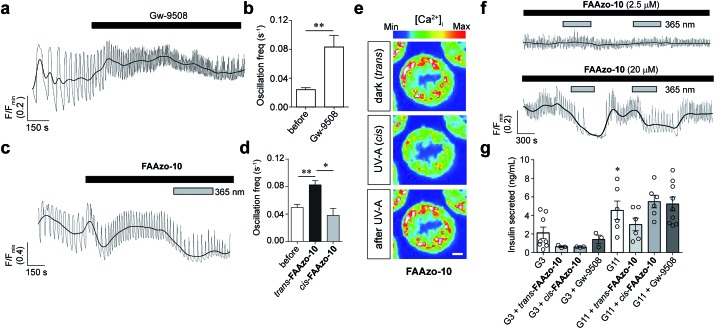
**FAAzo-10** enables optical control of [Ca^2+^]_i_ oscillations in pancreatic islets. [Ca^2+^]_i_ oscillations were stimulated by a high glucose concentration (11 mM, G11) and monitored in intact mouse islets using the fluorescent [Ca^2+^]_i_ indicator Fluo-8. (a, b) The application of Gw-9508 (50 μM) caused an increase in the [Ca^2+^]_i_ oscillation frequency. Displayed as (a) a representative trace from a single islet and (b) the oscillation frequency averaged over multiple islets (*n* = 6 recordings). (c, d) The application of *trans*-**FAAzo-10** (20 μM) also caused a marked increase in the oscillation frequency. Isomerization to *cis*-**FAAzo-10** with 365 nm irradiation reversed this effect. Results are displayed as (c) a representative trace from a single islet and (d) the average oscillation frequency from multiple islets (*n* = 5 recordings). (e, f) **FAAzo-10** enabled optical control of β-cell [Ca^2+^]_i_ oscillations at 20 μM, but not at 2.5 μM (*n* = 4–5 recordings) (representative images cropped to show a single islet; scale bar = 25 μm). (g) **FAAzo-10** (20 μM) did not afford a consistent effect on GSIS (3 mM glucose, G3). Gw-9508 (20 μM) also did not affect GSIS (*n* = 3–8 assays using islets from at least 3 animals) (* denotes significance between G3 and G11). Grey lines are raw traces (to show frequency effects), black lines are smoothed traces (to show amplitude effects). *P < 0.05 and **P < 0.01, ANOVA, with repeated measures as necessary. Error bars were calculated as ±s.e.m.

Finally, we evaluated our photoswitchable ligands for their effects on intact pancreatic islets using confocal fluorescence imaging. We employed the fluorescent small-molecule [Ca^2+^]_i_ indicator Fluo-8 to monitor [Ca^2+^]_i_ oscillations stimulated by a high glucose concentration (11 mM). Similar to the application of Gw-9508 (Fig. 7a and b), application of *trans*-**FAAzo-10** (20 μM) caused a marked increase in the [Ca^2+^]_i_ oscillation frequency (Fig. 7c and d). In line with the effects that would be expected from our results on GPR40, K_v_, and K_ATP_, isomerization to *cis*-**FAAzo-10** with 365 nm irradiation reversed this effect entirely (Fig. 7c–f). Lower concentrations of **FAAzo-10** (2.5 μM) did not affect oscillation frequency in either configuration (Fig. 7f). To exclude imaging artifacts, in particular fluorescence quenching, the cells were treated with a methyl ester FAAzo-derivative, **FAAzo-5(OMe)**, which possesses an azobenzene photoswitch with similar spectral characteristics to **FAAzo-10**.^30^**FAAzo-5(OMe)** produced a small increase in the [Ca^2+^]_i_ oscillation frequency in either configuration (Fig. S4a–c†), as methyl esterification of the acid group abolished *cis*-activity. Although **FAAzo-10** effectively increased [Ca^2+^]_i_ oscillations, we did not observe a significant increase in insulin secretion in either *trans* or *cis* at both low (3 mM) and high (11 mM) glucose concentrations (Fig. 7g). Similarly, benchmark Gw-9508 did not stimulate GSIS at 3 mM or 11 mM glucose (Fig. 7g). An effect of BSA on Gw-9508 and/or **FAAzo-10** potency was unlikely, since assays with low (3 mM) glucose concentration but performed in the absence of the carrier were identical (data not shown). Experiments were also repeated at high (17 mM) glucose, but without BSA, showing a similar lack of stimulation with Gw-9508 or **FAAzo-10** (Fig. S4d†). Neither **FAAzo-10** nor Gw-9508 were able to suppress tolbutamide-stimulated insulin secretion, further supporting an effect on K_ATP_ channel conductance (Fig. S4e†).^11^ UV-A irradiation alone did not affect oscillatory behavior or insulin secretion levels, as expected from previous studies^25^,^31^ (Fig. S4f†).

## Conclusions

In summary, we have demonstrated that **FAAzo-10** is a potent photoswitchable agonist of GPR40, and reversibly inactivates K^+^ channels in dissociated mouse β-cells. Although our previous studies using the FAAzos conjugated to different headgroups afforded *cis*-active compounds,^30^,^31^ we found the opposite in this case. **FAAzo-10** was more active in the *trans*-form at all targets, and can reversibly stimulate [Ca^2+^]_i_ oscillations in pancreatic β-cells using light. Interestingly, stimulation of [Ca^2+^]_i_ oscillations with **FAAzo-10** did not translate to increased insulin secretion in primary mouse islets, in line with the effects of the benchmark drug, Gw-9508. This suggests that oscillations by themselves are potentially not a sufficient signal for effective granule fusion, and that an additional factor was not triggered under these conditions. Of note, previous studies using Gw-9508 have afforded either stimulatory, inhibitory or no effect on insulin secretion,^11^,^21^,^39^ with two conflicting reports in mouse islets.^12^,^40^ As previously alluded to using the PhoDAGs,^31^ the variation of the effects induced by Gw-9508 application may stem from different protein expression levels or membrane area between immortalized and primary cells, or conversely off-target effects on GPR120, which shares some homology with GPR40. Similarly, differential effects caused by plasma membrane *vs.* intracellular fatty acid-signalling, as was observed using caged AA-derivatives, may contribute to this effect.^6^ By contrast, long chain fatty acids such as linoleic and palmitic acid have been consistently shown to potently stimulate insulin secretion, and this can be abrogated by GPR40 knockdown/silencing.^18^ Our studies thus reinforce the notion that signals in addition to GPR40 activation may be required for fatty-acid-stimulated insulin release, highlighting the complexity of fatty acid signalling in the β-cell, and underscoring the importance of **FAAzo-10** for studying the intricate relationship between [Ca^2+^]_i_ oscillations and insulin secretion. More broadly, **FAAzo-10** opens up the possibility to precisely interrogate the contribution of GPR40 signalling in different body compartments (*e.g.* brain and liver) to glucose homeostasis.

## Live subject statement

All studies were regulated by the Home Office per the Animals (Scientific Procedures) Act 1986 of the United Kingdom (PPL P2abc3a83), and study approval granted by the Animal Welfare and Ethical Review Body of the University of Birmingham.

## Conflicts of interest

The authors declare no conflicts of interest.

## Supplementary Material

Supplementary information
