# Supplementary material for: Optical control of GPR40 signalling in pancreatic β-cells
Source: Chem Sci. 2017 Aug 30;8(11):7604–10. doi: 10.1039/c7sc01475a (PMC5848828; doi:10.1039/c7sc01475a)
Supplement: Supplementary file 1 [file SC-008-C7SC01475A-s001.pdf]

Electronic Supplementary Material (ESI) for Chemical Science.
This journal is © The Royal Society of Chemistry 2017

Optical control of GPR40 signaling in pancreatic β-cells

James Allen Frank[1], Dmytro A. Yushchenko[2,3], Nicholas H.F. Fine[4,5,6], Margherita Duca[1,7], Mevlut Citir[2], Johannes Broichhagen[1,8], David J. Hodson*[5,6], Carsten Schultz*[2,9], Dirk Trauner*[1,10]

[1]Department of Chemistry and Center for Integrated Protein Science, Ludwig Maximilians University Munich, Butenandtstraße 5-13, 81377 Munich, Germany.

[2]European Molecular Biology Laboratory (EMBL), Cell Biology & Biophysics Unit, Meyerhofstraße 1, 69117 Heidelberg, Germany.

[3]Institute of Organic Chemistry and Biochemistry, Academy of Sciences of the Czech Republic, Flemingovo namesti 2, 16610 Prague 6, Czech Republic.

[5]Institute of Metabolism and Systems Research (IMSR), and Centre of Membrane Proteins and Receptors (COMPARE), University of Birmingham, Birmingham B15 2TT, UK.

[6]Centre for Endocrinology, Diabetes and Metabolism, Birmingham Health Partners, Birmingham, B15 2TH, UK.

[7]Department of Chemistry, University of Milan, Via Golgi 19, 20133, Milan, Italy.

[8]Max-Planck Institute of Medical Research, Jahnstr. 29, 69120 Heidelberg, Germany.

[9]Dept. of Physiology and Pharmacology, Oregon Health and Science University, Portland, OR 97237, USA.

[10]Department of Chemistry, New York University, 100 Washington Square East, New York, NY 10003-6699, USA.

*dirktrauner@nyu.edu

*schultz@embl-heidelberg.de

*d.hodson@bham.ac.uk

Supplementary Information

Supplementary Figures

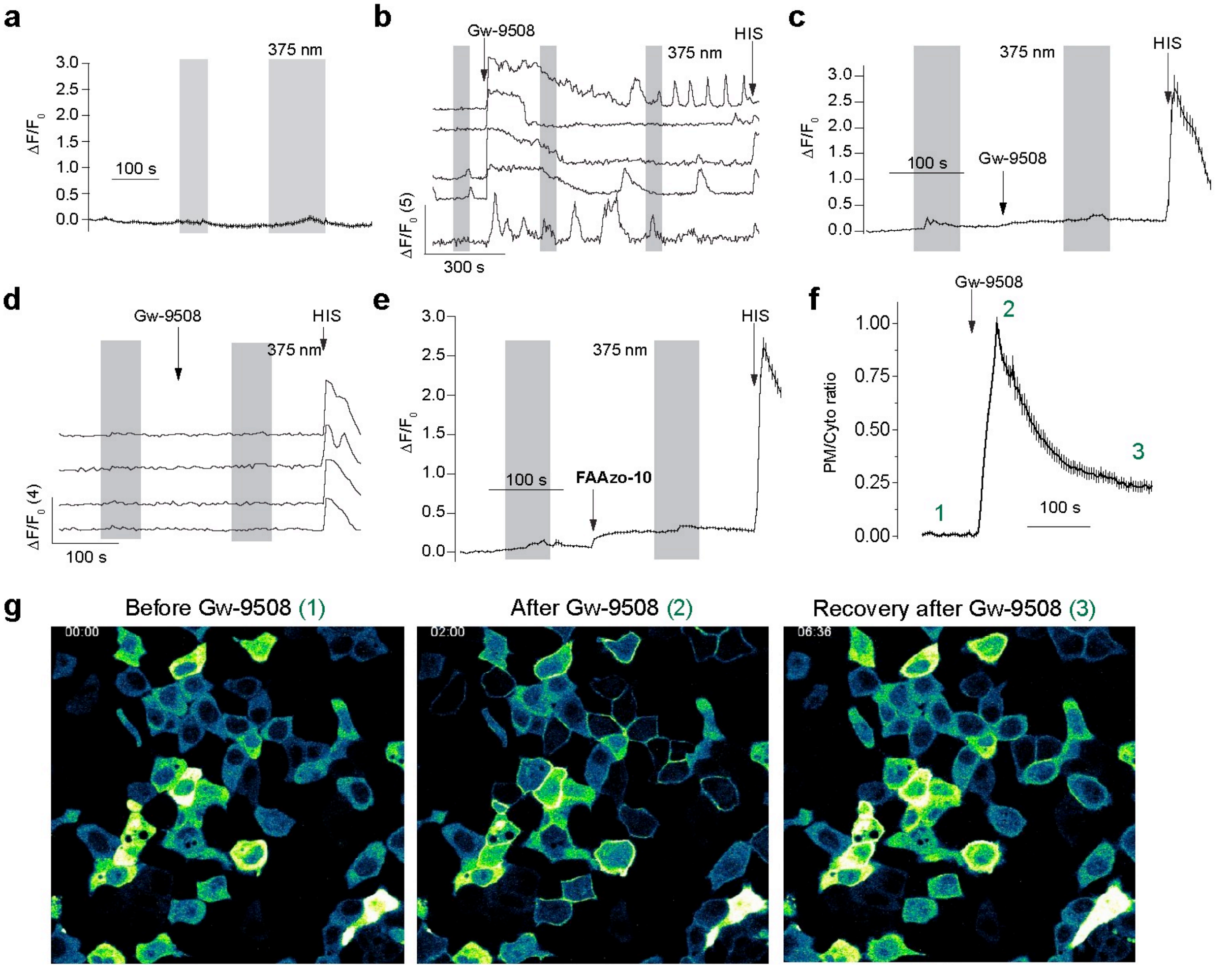

Figure S1 | Optical control of GPR40 in HeLa cells. $[Ca^{2+}]_i$ in HeLa cells was monitored using R-GECO. (**a**,**b**) When co-transfected with GPR40, 375 nm irradiation did not affect $[Ca^{2+}]_i$ (n = 95 cells from one experiment), and (**b**) application of Gw-9508 (200 nM) caused an increase in the $[Ca^{2+}]_i$ and oscillation frequency. This was not affected by 375 nm irradiation. (**c-e**) Without GPR40: (**c**,**d**) Gw-9508 (up to 20 μM) did not affect $[Ca^{2+}]_i$ levels. Displayed as (**c**) an average of many cells (n = 65 cells from one experiment) and (**d**) individual traces from representative cells. (**e**) **FAAzo-10** (200 nM) also did not affected $[Ca^{2+}]_i$ levels. Displayed as the average $[Ca^{2+}]_i$ level from many cells (n = 159 cells from two experiments). HIS (10 μM) was used as a positive control to induce $[Ca^{2+}]_i$. (**f**) Gw-9508 (200 nM) triggered the translocation of C1-GFP towards the plasma membrane on application. Averaged response of 32 cells. (**g**) Confocal images of HeLa cells expressing GPR40, C1-GFP and R-GECO illustrating translocation if C1-GFP after addition of GW-9508, as described in (**f**). Error bars were calculated as ±s.e.m.

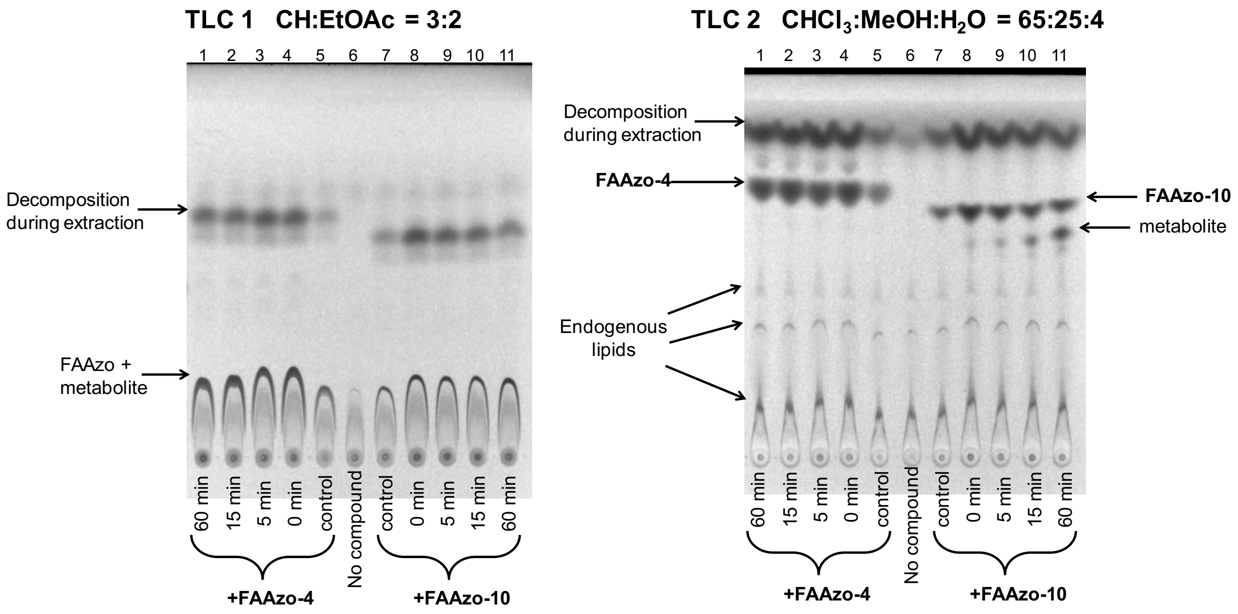

Figure S2 | FAAzo metabolism in HeLa Kyoto cells. TLCs of: **FAAzo-4** (lane 5) and **FAAzo-10** (lane 7) spiked into DMSO-treated HeLa cells at the beginning of lipid extraction (controls), endogenous lipids extracted from DMSO-treated HeLa cells (no **FAAzos**) (lane 6), lipids extracted from HeLa cells which were loaded (for 5 min) with 100 μM **FAAzo-4** (lanes 1-4) or **FAAzo-10** (lanes 8-11) and incubated with corresponding FAAzo over indicated time intervals. A cyclohexane/EtOAc system was used to elute the TLC 1. Thus, this system is suitable for determining the presence of molecules that are more lipophilic than FAAzos and have higher retention factor (R_f) than FAAzos which run in this system with R_f ~0.22-0.25. TLC 1 demonstrates that during up to 1 h incubation of FAAzos in cells, they do not form more lipophilic metabolites, such as diacylglycerol or triacylglycerol derivatives, as there is no appearance of new bands with R_f higher than those of FAAzos (except the bands that correspond to decomposition of FAAzos during extraction). A $CHCl_3$/MeOH/H_2O system used to elute the TLC 2 is more suitable for determining the presence of less lipophilic molecules than FAAzos, such as phosphatidic acids or PIPs. TLC 2 demonstrates that **FAAzo-4** is not metabolized even after 1 h incubation in cells. Meanwhile metabolism of **FAAzo-10** leads to formation of less lipophilic metabolites, as there is appearance of the band on the TLC 2 with R_f = 0.6 (just below the band corresponding to **FAAzo-10**, R_f = 0.67) intensity of which increases with increase of **FAAzo-10** incubation time in cells. However even after 1 h incubation, >50% of non-metabolized **FAAzo-10** remained in cells.

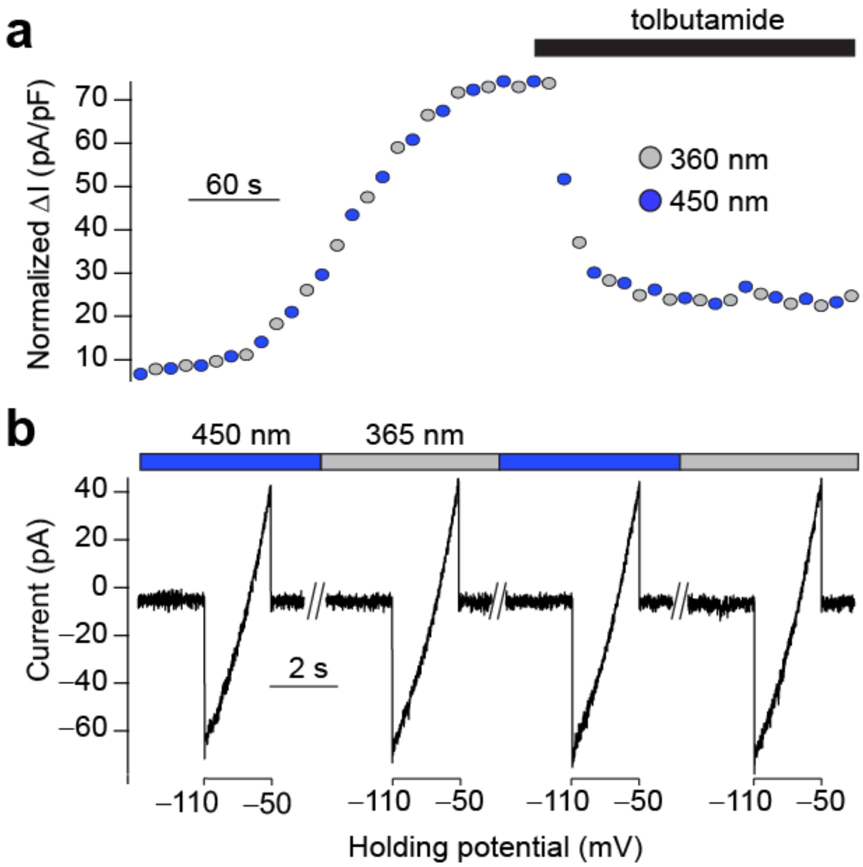

Figure S3 | Control experiments for K_{ATP} patch-clamp experiments. K_{ATP} currents were recorded from primary mouse β-cells using whole-cell electrophysiology. (**a**) After break-in, the K_{ATP} current developed as the cytoplasm exchanged with the pipette solution. Tolbutamide (40 μM) reduced the magnitude of the K_{ATP} current. Plotted is the ΔI across a voltage ramp (over 2 s, every 10 s) from −110 to −50 mV under alternating 350 and 450 nm irradiation, from a representative cell. (**b**) UV-A and blue irradiation alone did not affect the K_{ATP} conductance, displayed as sequential voltage ramps under alternating UV-A and blue light from a representative cell.

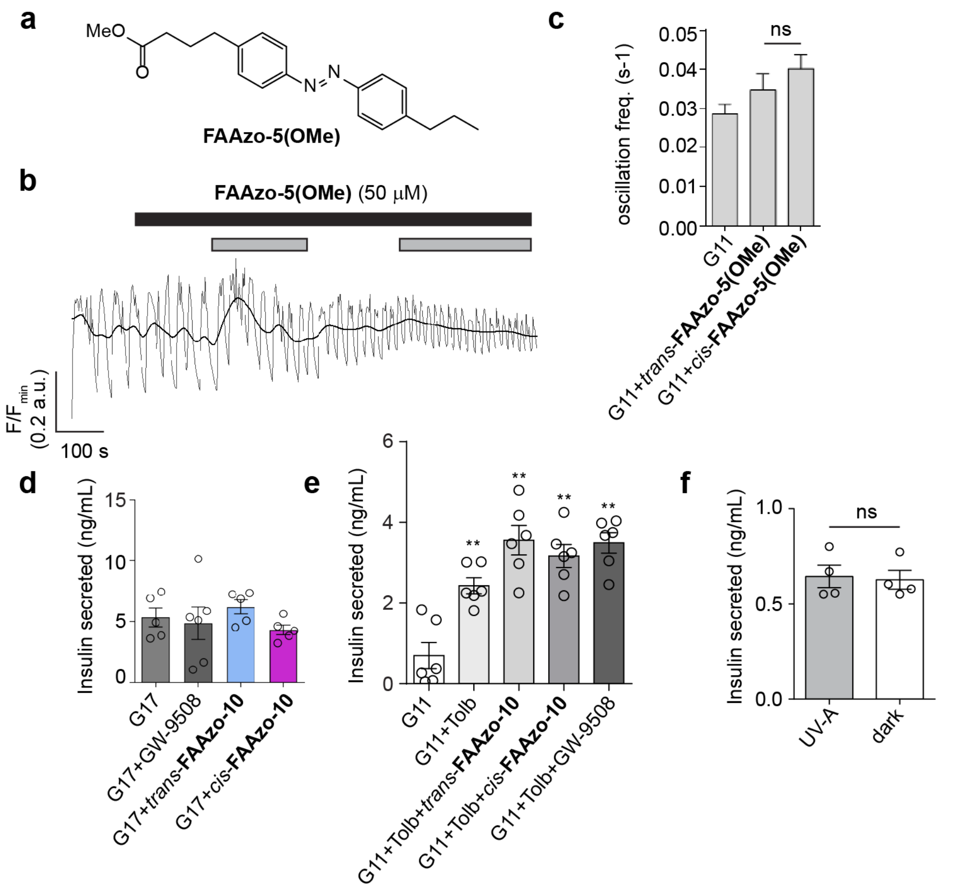

Figure S4 | Control experiments in mouse islets. (**a**) The chemical structure of **FAAzo-5(OMe)**[1]. (**b**,**c**) $[Ca^{2+}]_i$ from isolated mouse islets were monitored using Fluo-8. Application of **FAAzo-5(OMe)** (50 μM) increased glucose-stimulated (11 mM) Ca^{2+} oscillation frequency, but this was not suppressed by UV-A illumination (n = 12 recordings). Displayed as (**b**) a representative trace from a single islet and (**c**) the averaged oscillation frequency from multiple islets in the presence of *trans*- and *cis*-**FAAzo-5(OMe)** (n = 12 recordings). Grey lines are raw traces (to show frequency effects), black lines are smoothed traces (to show amplitude effects). (**d**) Neither **FAAzo-10** (20 μM) nor Gw-9508 (20 μM) were able to influence insulin secretion in the presence of high (17 mM) glucose without BSA (n = 2 assays using islets from at least 3 animals). (**e**) Neither *cis/trans*-**FAAzo-10** nor Gw-9508 were able to suppress tolbutamide (Tolb, 100 μM)-induced insulin secretion (11 mM glucose). Significance was calculated compared to G11-alone. (**f**) UV-irradiation did not affect insulin secretion from islets in the presence of 3 mM glucose. Error bars were calculated as ±s.e.m. ns = not significant = P>0.05, **P<0.01.

Cell Culture

HeLa Kyoto cells were grown in 1.0 g/L D-glucose DMEM (GIBCO, cat # 31885-023) supplied with 10% FBS (GIBCO, cat # 10270-106) and 0.1 mg/mL antibiotic Primocin (Invitrogen, ant-pm-1). HeLa cells were first seeded in an 8-well Lab-Tek™ chambered coverslip (ThermoScientific #155411) 24-48 h before transfection at 37 °C and 5% CO_2. Transfection was carried out with FugeneHD (Promega, cat # E2311) in DMEM free of FBS and antibiotics according to the manufacturer's instructions. First, the media was aspirated and the wells were charged with DMEM media (200 μL per well). A transfection solution containing DMEM (20 μL per well), cDNA (300 ng total DNA per well) and FugeneHD (1.5 μL per well) was then added to each well of the 8-well Lab-Tek™. The cells were incubated at 37 °C and 5% CO_2 for 20-24 h before the microscopy experiments were performed.

Culture of primary mouse pancreatic islets

Islets were isolated from C57BL6 and CD1 mice using collagenase digestion, as previously detailed[2]. Briefly, following euthanasia by cervical dislocation, the bile duct was injected with a collagenase solution (1 mg/mL) before digestion at 37 °C for 10 min and separation of islets using a Histopaque gradient (1.083 and 1.077 g/mL). Islets were cultured for 24-72 h in RPMI medium supplemented with 10% FCS, 100 U/mL penicillin and 100 μg/mL streptomycin.

List of utilized cDNA constructs

Name	Characterization
R-GECO[3]	red intensiometric $[Ca^{2+}]_i$ sensor
*h*GPR40 (FFAR1)*	human free fatty acid receptor 1
C1-GFP[4]	green fluorescent DAG-sensing translocation probe

**This cDNA clone was obtained from the cDNA Resource Center (*www.cdna.org*).*

Laser scanning confocal microscopy

Imaging of HeLa cells was performed on an Olympus Fluoroview 1200 with a 20x objective, or a 63x oil objective. C1-GFP excitation was performed with 488 nm laser at low laser power (<3%) and emission was collected at 500-550 nm. R-GECO excitation was performed with a 559 nm laser at low laser power (<3%) and emission was collected at 570-670 nm. Compound activation was triggered using the quench function in the Olympus software. Photoactivation was carried out with a 375 nm laser at 100% intensity. The cells were incubated in imaging buffer (250 μL, containing in mM: 115 NaCl, 1.2 $CaCl_2$, 1.2 $MgCl_2$, 1.2 K_2HPO_4, 20 HEPES, 20 D-glucose) at 37 °C and 5% CO_2 for at least 10 min. Compounds were first solubilized in DMSO at a concentration of 10 mM. This stock was then diluted into imaging buffer (50 μL) and then added directly to the well containing the cells and imaging buffer.

Imaging of primary rodent pancreatic β-cells was performed using a Crest X-Light Nipkow spinning disk head coupled to a Nikon Ti-E automated base and 10x objective. Dye excitation was performed at 470/24 nm using a Lumencor Spectra X Light engine, and emitted signals captured at 500–550 nm using a Photometrics Evolve Delta 512 EMCCD. For photoswitching, irradiation was applied at 395/25 nm using the Lumencor source.

A small number of experiments were carried out using a Zeiss Axiovert M200 coupled to a Yokogawa CSU10 spinning disk head and a 10x objective. Fluo-8 excitation was performed using a solid-state 491 nm laser, and emission collected using a Hammamatsu C9100-13 EM-CCD at 500-550 nm. Photoactivation was carried out using an X-Cite 120 epifluorescence source and a 350 ± 20 nm band-pass filter. In all cases, islets were imaged in a HEPES-bicarbonate buffer containing (in mM): 120 NaCl, 4.8 KCl, 24 $NaHCO_3$, 0.5 Na_2HPO_4, 5 HEPES, 2.5 $CaCl_2$, 1.2 $MgCl_2$ and 11 D-glucose. Images were processed with Fiji software (http://fiji.sc/Fiji) and the resulting data was analyzed in Microsoft Excel, MATLAB and R. The data were then plotted with Igor Pro, Origin and/or R.

Whole-cell electrophysiology in dissociated mouse β-cells

Two days after culture, primary mouse islets were dissociated into single β-cells using trypsin digestion for 5 min at 37 °C and allowed to attach to poly-L-lysine-coated and acid-etched coverslips overnight in RPMI medium supplemented with 10% FCS, 100 U/mL penicillin and 100 μg/mL streptomycin at 37 °C and 5% CO_2. Whole cell patch clamp experiments were performed the next day using a standard electrophysiology setup equipped with a HEKA Patch Clamp EPC10 USB amplifier and PatchMaster software (HEKA Electronik). Micropipettes were generated from “Science Products GB200-F-8P with filament” pipettes using a Narishige PC-10 vertical puller. The patch pipette resistance varied between 4-8 MΩ.

For recording of the K_v current, the bath solution contained (in mM): 119 NaCl, 2 $CaCl_2$, 4.7 KCl, 10 HEPES, 1.2 $MgSO_4$, 1.2 KH_2PO_4, 14.4 D-glucose (adjusted to pH 7.3 with NaOH). The intracellular solution contained (in mM): 140 KCl, 1 $MgCl_2$, 10 EGTA, 10 HEPES, 5 MgATP (adjusted to pH 7.25 with KOH). In voltage clamp mode, voltage steps (500 ms) were applied to the cells from the baseline at –70 mV to +80 mV in 10 mV intervals. The resulting current was averaged between the 400-470 ms range of each voltage step and plotted as a function of the holding potential.

For measurement of the K_{ATP} current, the bath solution contained (in mM): 116 NaCl, 3 KCl, 4 $CaCl_2$, 3 $MgCl_2$, 25 HEPES (adjusted to pH 7.4 with NaOH). The intracellular solution contained (in mM): 130 K-gluconate, 10 KCl, 10 NaCl, 3 $MgCl_2$, 4 EGTA, 10 HEPES, 0.3 MgATP (adjusted to pH 7.2 with KOH). In voltage clamp mode, voltage steps (500 ms) were applied to the cells from the baseline at −110 mV to −50 mV in 10 mV intervals. The resulting current was averaged between the 100-450 ms range of the voltage step and plotted as a function of the holding potential. All cells had a leak current below 15 pA on break-in at –70 mV. Recordings were corrected for the liquid junction potential. The data was analyzed in Igor Pro using the Patcher`s Power Tools (MPI Göttingen) plugin. Current values were extrapolated and processed in Igor Pro and the Microscoft Excel, and the results were again plotted in Igor Pro.

Quantification of insulin secretion

Insulin secretion was measured using static incubation of 8 islets in low-bind Eppendorf's for 30 min at 37 °C in Krebs-HEPES bicarbonate solution containing (in mM): 130 NaCl, 3.6 KCl, 1.5 $CaCl_2$, 0.5 $MgSO_4$, 0.5 NaH_2PO_4, 2 $NaHCO_3$, 10 HEPES and 0.1% (wt/vol) bovine serum albumin, pH 7.4[5]. A subset of experiments was repeated without BSA. Treatments were applied as indicated, and photoswitching performed at 365 ± 40 nm using either a UV tube lamp placed under the samples or pre-illumination of compound at 340-380 nm using higher power LEDs. Insulin concentration secreted into the supernatant was determined using a homogeneous time-resolved fluorescence (HTRF) assay (Cisbio), according to the manufacturer's instructions.

TLC analysis

Hela Kyoto cells were grown to ~90% confluency in DMEM high glucose (suppl. w/ 10% FBS, 4 mM L-glutamine) in 6 cm dishes at 37 °C and 5% CO_2, washed 2 times with imaging buffer containing (in mM): 140 NaCl, 2.5 KCl, 1.8 $CaCl_2$, 1.0 $MgCl_2$, 20 HEPES (pH 7.4, freshly supplemented with 0.4% w/v D-glucose and 2 mM L-glutamine). After aspirating the buffer from the dishes, previously prepared mixtures of **FAAzo-4** or **FAAzo-10** (15 μL of 10 mM lipid in DMSO solution was diluted in 1.5 mL of imaging buffer and vortexed) were added to the dishes (final concentration 100 μM). The cells were loaded with the corresponding compound for 5 min, then washed again 3 times with imaging buffer and further incubated (without extracellular compound) in imaging buffer for indicated time intervals (0 min, 5 min, 15 min, 60 min).

The cells were washed again with PBS (w/o Ca^{2+}, Mg^{2+}) twice and treated with trichloroacetic acid (1 mL of 0.5 M) for 5 min on ice. After that the cells were scraped and centrifuged at 20,000g for 3 min. The pelleted material was washed twice with 5% (w/v) trichloroacetic acid supplemented by EDTA (10 mM), and the resulting pellet was subjected to lipid extraction with 750 μL of MeOH:$CHCl_3$ (2:1).

After 10 min continuous shaking on an Eppendorf Thermomixer and then centrifugation, the supernatant was transferred to another vial and mixed further with 750 μL of $CHCl_3$ and 375 μL aqueous HCl (1 M). Phase separation was obtained by centrifugation at 1000g for 5 min, and then the lower phase was collected and evaporated. The obtained lipid extract was solubilized in $CHCl_3$ and the obtained solution was divided into two equal parts which were spotted on two HPTLC silica gel plates F254 (Merck Millipore). TLC 1 was eluted with

cyclohexane:EtOAc (3:2 vol:vol) system. TLC 2 eluted with $CHCl_3$:MeOH:H_2O (65:25:4 vol:vol:vol).

Images were obtained by a Bio-Rad Chemidoc Touch Imaging System using the EtBr channel. We took advantage of the F254 fluorescence quenching by the azobenzenes on the TLC plate.

Data reporting and error analysis

For fluorescence imaging experiments, “n” is the number of individual cells measured. The number of independent trials included in each panel is also included in each figure caption. Ca^{2+} traces were normalized as either: F/F_{min}, where F = fluorescence intensity at any given timepoint and F_{min} = minimum fluorescence intensity for the recording; or $\Delta F/F_0$, where F_0 is the fluorescence intensity at t = 0 s, and ΔF is the difference in fluorescence intensity at that timepoint time from the intensity at F_0. Both F/F_{min} and $\Delta F/F_0$ are unitless ratios. For single cell $[Ca^{2+}]_i$ traces, the magnitude of this value is represented in brackets on the vertical scale bar. For patch-clamp experiments, “n” represents the number of individual cells included in the dataset. For insulin quantification, “n” represents the number of independent experiments performed. In all cases, averaged data is plotted as mean ±s.e.m., using "n" as the number of samples. For pairwise comparisons, either Student’s t-test or the Mann-Whitney test were used to determine statistical significance. Where multiple comparisons were considered, normal or repeated measures one-way ANOVA was used followed by either Bonferroni’s or Sidak’s post-hoc tests.

General synthetic procedures

FAAzo-4 was prepared as previously described[1]. All reagents and solvents were purchased from commercial sources (Sigma-Aldrich, TCI Europe N.V., Strem Chemicals, etc.) and were used without further purification unless otherwise noted. Dry solvents were purchased from Acros Organics as "extra dry" reagents and used as received. Reactions were monitored by TLC on pre-coated, Merck Silica gel 60 F_{254} glass-backed plates and the chromatograms were first visualized by UV irradiation at 254 nm, followed by staining with ceric ammonium molybdate solution (CAM) and finally gentle heating with a heat gun. Flash silica gel chromatography was performed using silica gel (SiO_2, particle size 40-63 μm) purchased from Merck.

All NMR spectra were measured on a BRUKER Avance III HD 400 (equipped with a CryoProbe™). Multiplicities in the following experimental procedures are abbreviated as follows: s = singlet, d = doublet, t = triplet, q = quartet, quint = quintet, sext = sextet, hept = heptet, br = broad, m = multiplet. ^{1}H chemical shifts are expressed in parts per million (ppm, δ scale) and are referenced to the residual protium in the NMR solvent ($CDCl_3$: δ = 7.26). ^{13}C chemical shifts are also expressed in ppm (δ scale) and are referenced to the carbon resonance of the NMR solvent ($CDCl_3$: δ = 77.16). NOTE: Due to the *trans/cis* isomerisation of some compounds containing an azobenzene functionality, more signals are observed in the ^{1}H and ^{13}C spectra than would be expected for the pure *trans*-isomer. Only signals for the major *trans*-isomer are reported, however the identities of the remaining peaks were verified by 2D-COSY, HSQC and HMBC experiments.

UV-Vis spectra were recorded using a Varian Cary 50 Bio UV-Visible Spectrophotometer with Helma SUPRASIL precision cuvettes (10 mm light path). Switching was achieved using a Polychrome V (Till Photonics) monochromator. The illumination was controlled using PolyCon3.1 software and the light was guided through a fiber-optic cable with the tip pointed directly into the top of the sample cuvette.

Infrared (IR) spectra were recorded as neat materials on a PERKIN ELMER Spectrum BX-59343 instrument. For detection, a SMITHS DETECTION DuraSam-plIR II Diamond ATR sensor was used. The measured wave numbers are reported in cm^{-1}.

Low- and high-resolution electron ionization (EI) mass spectra were obtained on a MAT CH7A mass spectrometer. Low- and high-resolution electrospray ionization (ESI) mass spectra were obtained on a Varian MAT 711 MS instrument operating in either positive or negative ionization modes.

Melting points were measured on a Stanford Research Systems MPA120 EZ-Melt apparatus in open glass capillaries.

Compound synthesis and characterization

3-(4-((3-Phenoxyphenyl)diazenyl)phenyl)propanoic acid (FAAzo-10)

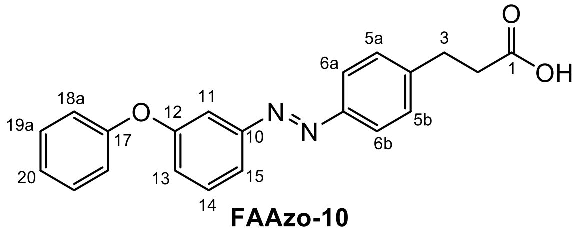

FAAzo-10

3-phenoxyaniline (112 mg, 0.600 mmol, 2.0 equiv.) was dissolved in CH_2Cl_2 (25 mL), and then an aqueous solution (25 mL) of Oxone® (372 mg, 1.20 mmol, 4.0 equiv.) was added. The biphasic mixture was stirred rapidly overnight. The phases were then separated and the organic phase was washed with H_2O (2x30 mL), dried over anhydrous Na_2SO_4 and filtered. The filtrate was concentrated under reduced pressure and the residue was purified by flash silica gel chromatography (10 g SiO_2, CH_2Cl_2). The fractions containing the green 1-nitroso-3-phenoxybenzene were pooled and 3-(4-aminophenyl)propionic acid (50 mg, 0.30 mmol, 1.0 equiv.) was added alongside AcOH (20 mL). The solution was again stirred overnight at room temperature. The solvents were removed under reduced pressure and the crude residue was purified by flash silica gel chromatography (10 g SiO_2, 7:3 hexane:EtOAc with 1% AcOH) to yield **3-(4-((3-phenoxyphenyl)diazenyl)phenyl)propanoic acid** (**FAAzo-10**, 46.7 mg, 45%) as an orange solid.

TLC (70:30:1 hexane:EtOAc:AcOH): R_f = 0.5 (*trans*), 0.36 (*cis*).

^{1}H NMR ($CDCl_3$, 400 MHz, 25 °C): δ 11.33 (bs, 1 H, H_{COOH}), 7.85 (d, 2 H, $H6_{a,b}$, J = 8.0 Hz), 7.68 (d, 1 H, H15, J = 7.8 Hz), 7.56 (s, 1 H, H11), 7.48 (t, 1 H, H14, J = 8.0 Hz), 7.41-7.33 (m, 4 H, $H5_{a,b}$, $H19_{a,b}$), 7.18-7.14 (m, 2 H, H13, H20), 7.10 (d, 2 H, $H18_{a,b}$, J = 8.0 Hz), 3.04 (t, 2 H, $H3_{a,b}$, J = 7.8 Hz), 2.74 (t, 2 H, $H2_{a,b}$, J = 7.6 Hz).

^{13}C NMR ($CDCl_3$, 101 MHz, 25 °C): δ 179.1 (C1), 158.2 (C_{Azo}), 156.9 (C_{Azo}), 154.2 (C_{Azo}), 151.3 (C_{Azo}), 143.8 (C4), 130.2 (C14), 130.0 (2 C, $C19_{a,b}$), 129.2 (2 C, $C5_{a,b}$), 123.8 (C20), 123.3 (2 C, $C6_{\mathbf{a,b}}$), 121.2 (C13), 119.3 (2 C, $C18_{\mathbf{a,b}}$), 118.6 (C15), 112.1 (C11), 35.4 (C2), 30.5 (C3).

IR (neat, ATR): ṽ (cm^{-1}) = 3038, 2924, 2623, 1694, 1602, 1586, 1488, 1437, 1417, 1314, 1277, 1253, 1215, 1166, 1150, 1104, 1072, 1022, 963, 943, 916, 899, 865, 838, 792, 779, 749, 680.

HRMS (ESI$^+$): *m/z* calcd. for $[C_{21}H_{19}N_2O_3]^+$: 347.1396, found: 347.1390 ($[M+H^+]^+$).

UV-Vis (50 μM in DMSO): $\lambda_{max}(\pi-\pi^*)$ = 330 nm. $\lambda_{max}(n-\pi^*)$ = 430 nm.

Melting point (°C): 129.8-132.0.

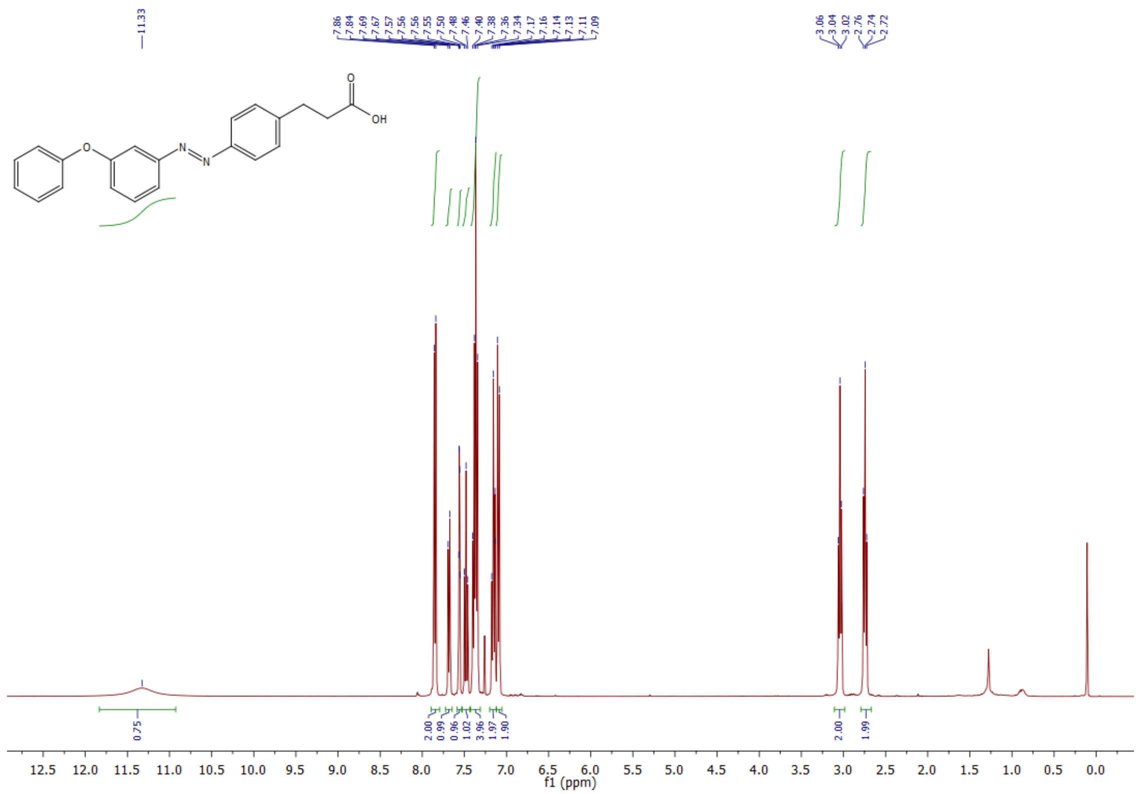

11.33
7.86
7.84
7.69
7.67
7.57
7.56
7.56
7.55
7.50
7.48
7.46
7.40
7.38
7.36
7.34
7.17
7.16
7.14
7.13
7.11
7.09
3.06
3.04
3.02
2.76
2.74
2.72
O
OH
O
N
N
0.75
2.00
0.99
0.96
1.02
3.96
1.97
1.90
2.00
1.99
f1 (ppm)

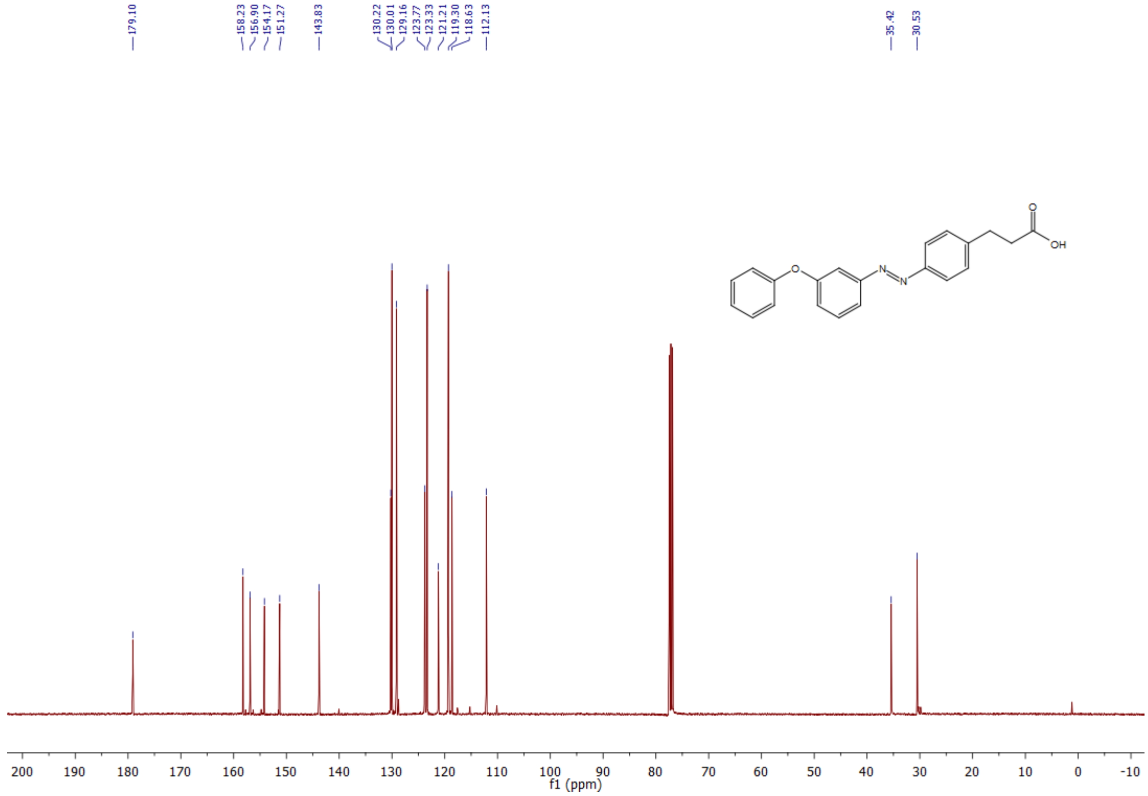

179.10
158.23
156.90
154.17
151.27
143.83
130.22
130.01
129.16
123.77
123.33
121.21
119.30
118.63
112.13
35.42
30.53
O
OH
O
N
N
f1 (ppm)

References

1 J. A. Frank, M. Moroni, R. Moshourab, M. Sumser, G. R. Lewin and D. Trauner, *Nat. Commun.*, 2015, **6**, 7118.

2 A. Ravier, Magalie and A. Rutter, Guy, in *Mouse Cell Culture*, 2010, vol. 633, pp. 171–184.

3 Y. Zhao, S. Araki, J. Wu, T. Teramoto, Y.-F. Chang, M. Nakano, A. S. Abdelfattah, M. Fujiwara, T. Ishihara, T. Nagai and R. E. Campbell, *Science*, 2011, **557**, 1888–1891.

4 E. Oancea, M. N. Teruel, A. F. G. Quest and T. Meyer, *J. Cell Biol.*, 1998, **140**, 485–498.

5 M. A. Ravier and G. A. Rutter, *Diabetes*, 2005, **54**, 1789–1797.
